# Mass spectrometric identification and toxicity assessment of degraded products of aflatoxin B1 and B2 by *Corymbia citriodora* aqueous extracts

**DOI:** 10.1038/srep14672

**Published:** 2015-10-01

**Authors:** Wajiha Iram, Tehmina Anjum, Mazhar Iqbal, Abdul Ghaffar, Mateen Abbas

**Affiliations:** 1 Institute of Agricultural Sciences, University of the Punjab, Pakistan; 2 Health Biotechnology Division, National Institute for Biotechnology & Genetic Engineering (NIBGE), Faisalabad, Pakistan; 3 Department of Chemistry University of Engineering and Technology Lahore, Pakistan; 4 Quality Operating Laboratory (QOL), University of Veterinary and Animal Sciences, Pakistan

## Abstract

This study explores the detoxification potential of *Corymbia citriodora* plant extracts against aflatoxin B1 and B2 (AFB1; 100 μg L^−1^ and AFB2; 50 μg L^−1^) in *In vitro* and *In vivo* assays. Detoxification was qualitatively and quantitatively analyzed by TLC and HPLC, respectively. The study was carried out by using different parameters of optimal temperature, pH and incubation time period. Results indicated that *C. citriodora* leaf extract(s) more effectively degrade AFB1 and AFB2 i.e. 95.21% and 92.95% respectively than *C. citriodora* branch extract, under optimized conditions. The structural elucidation of degraded toxin products was done by LCMS/MS analysis. Ten degraded products of AFB1 and AFB2 and their fragmentation pathways were proposed based on molecular formulas and MS/MS spectra. Toxicity of these degraded products was significantly reduced as compared to that of parent compounds because of the removal of double bond in the terminal furan ring. The biological toxicity of degraded toxin was further analyzed by brine shrimps bioassay, which showed that only 17.5% mortality in larvae was recorded as compared to untreated toxin where 92.5% mortality was observed after 96hr of incubation. Therefore, our finding suggests that *C. citriodora* leaf extract can be used as an effective tool for the detoxification of aflatoxins.

Aflatoxins (AFs) are group of potent mycotoxins with mutagenic, carcinogenic, teratogenic, hepatotoxic and immunosuppressive properties, are of particular importance because of their major occurrence and adverse effects on animal and human health^1^,^2^. The Food and Agriculture Organization (FAO) estimated that many basic foods could be contaminated by the mycotoxin producing fungi, which contributes to enormous global losses of food, approximately 1000 million metric tons each year^3^. Among 18 different types of aflatoxins identified, the major members are aflatoxin B1, B2, G1 and G2 which chemically are coumarin derivatives with a fused dihydrofurofuran moiety. *Aspergillus flavus* produces AFB1 and AFB2, whereas, *Aspergillus parasiticus* produces AFB1, AFB2, AFG1 and AFG2^4^,^5^. Among them, AFB1 has the greatest potential as an environmental carcinogen, with toxic effects on human via its direct or indirect consumption in food products^6^. The European Union has enacted a very stringent aflatoxin tolerance threshold of 2 μg/kg aflatoxin B1 and 4 μg/kg total aflatoxins for nut and cereals for human consumption^7^. Since aflatoxins can cause acute, subacute and chronic toxicity in animals and human, much emphasis has been focused on the control or elimination of these toxic metabolites in food grains and livestock feeds^8^.

Detoxification of aflatoxins appears to be a more attractive approach. Detoxification strategies have been arbitrarily divided into physical, chemical, or microbiological processes to detoxify by destroying, modifying, or absorbing the mycotoxin so as to reduce or eliminate the toxic effects^9^. However, each treatment has its own limitations. During physical methods of aflatoxin detoxification like cooking and roasting various nutrients are destroyed from treated food commodity^9^,^10^. While chemicals methods like ammoniation, treatment with formaldehyde and sodium bisulphite have been found to be effective in detoxification of aflatoxins but their use in food industry is restricted because of food safety issues^11^. Biological detoxification of aflatoxins by employing microorganisms have been demonstrated by several researchers with major drawback of utilizing nutrients from food for their own growth and multiplication and release of undesirable compounds^12^,^13^. So there is a need to identify biologically safe and cost effective aflatoxin detoxifying compounds for use in food and feed industries. Since the treated product should be safe and unaffected by the chemicals used and the nutritive values of the treated product should not be altered.

Natural plant products may provide alternative way to prevent food or feed from fungal or mycotoxin contamination. Powder and extract of many medicinal herbs and higher plants have been reported to inhibit the growth of toxigenic fungi and production of toxins^14^,^15^. Reddy *et al*.^16^ investigated the potential of certain plant extracts and biocontrol agents for the reduction of aflatoxin B1 (AFB1) in stored rice. Among the plant extracts tested, *Syzigium aromaticum* (L.) Merr. Et Perry, *Curcuma longa* (L.), *Allium sativum* L. and *Ocimum sanctum* (Linn.) effectively inhibited the *A. flavus* growth and AFB1 production. Similarly, Velazhahan *et al*.^17^ evaluated various medicinal plants extracts for their ability to detoxify aflatoxin G1 (AFG1) by thin-layer chromatography and enzyme-linked immunosorbent assay (ELISA). Of the various plant extracts, the seeds extract of *Trachyspermum ammi* showed maximum degradation of AFG1. Another study by Vijayanandraj *et al*.^18^ also demonstrated the effect of different parameters on aqueous extracts of various medicinal plants for detoxification of aflatoxin B1 (AFB1). They found that leaf extracts of *Adhatoda vasica* Nees showed 98% degradation of AFB1 after incubation for 24h at 37 °C. Correspondingly, Kannan and Velazhahan^19^ explored the potential of some indigenous medicinal plants extracts for detoxification of aflatoxins. Their study showed that among various tested plants, *Barleria lupulina* Lindl. leaf extract(s) exhibited maximum detoxification of aflatoxin B1, B2, G1 and G2 at pH 10 whereas detoxification percentage decreased at pH 7 and 3. Time course study of aflatoxin detoxification by *B. lupulina* extract showed that degeneration of aflatoxin occurred within 10 min and this percentage was increased with increase in incubation period. In this study *Corymbia citriodora* aqueous extract is used which has been reported to possess known antibacterial, antifungal, anti-tumor, antioxidant, analgesic and anti-inflammatory effects by various researchers. So we use this plant to explore its potential to detoxify aflatoxins by developing a cost effective and an eco-friendly strategy for detoxification.

## Results and Discussion

In the present study the aqueous extracts of *Corymbia citriodora* leaf and branch were evaluated for their ability to detoxify AFB1 and AFB2. Degradation capability of plant extracts were qualitatively analyzed by TLC, which exhibited that in the presence of *C. citriodora* leaf extract(s), as compared to *C. citriodora* branch extract, fluorescence of recovered AFB1 and AFB2 was very weak and more incubation lead to more distinct decline in fluorescence. While no loss of fluorescence was observed in toxin recovered from untreated control samples. Detoxification was quantitatively analyzed by HPLC. Results showed that maximum degradation of AFB1 and AFB2 was observed in *C. citriodora* leaf extract(s) i.e. 95.21% and 92.95% respectively.

### Effect of temperature and incubation period on toxin detoxification by plant extracts (*In Vitro*)

Toxin detoxification was conducted at different temperatures for 3, 6, 12, 24, 48 and 72 hr of incubation (Table 1). The extent of detoxification was compared with control under same conditions. Time course study of toxin degradation showed that detoxification of AFB1 and AFB2 occurred within 3 hrs and percentage of degradation gradually increased with increase in incubation time. Maximum degradation was observed after 72 hrs of incubation. Similar findings were also observed by earlier workers (Hajare *et al*.^20^; Velazhahan *et al*.^17^; Vijayanandraj *et al*.^18^; Kannan and Velazhahan^19^).

In case of temperature, highest inactivation was observed at 60 °C. At this temperature, respective control (water) showed 13.36% and 8.64% detoxification of AFB1 and AFB2 after 72 hrs of incubation, respectively. However, toxin treated with *C. citriodora* leaf and branch extracts showed 99.56% and 85.21% detoxification of aflatoxin B1 while detoxification of aflatoxin B2 was 92.20% and 69.89% respectively, under same conditions. This detoxification may be due to synergistic effect of heat and moisture^15^,^16^. Similarly, Hajare *et al*.^20^ worked on aflatoxin inactivation by using Ajwain seeds extract under optimized conditions. According to his findings, highest inactivation was observed at 60 °C but further studies were conducted on 45 °C to eliminate the effect of heat and moisture on toxin inactivation.

In the present study, only 4.19% and 3.41% of AFB1 and AFB2 was found to be inactivated in control samples at 30 °C. While in treated samples at 30 °C, *C. citriodora* leaf extract(s) exhibited higher degree of AFB1 and AFB2 detoxification i.e., 82.55% and 83.27% than *C. citriodora* branch extract which showed 73.50% and 60.96% detoxification of AFB1 and AFB2, respectively (Table 1). Hence, further studies were conducted only at 30 °C after 72 hrs of incubation in order to eliminate the effects of external factors on toxin detoxification.

### Effect of pH on toxin detoxification by plant extracts (*In Vitro*)

Studies on the effect of pH on detoxification of aflatoxins by Corymbia plant extracts revealed that maximum detoxification was observed at pH 10 followed by pH 8. Results showed that *C. citriodora* leaf extract(s) significantly detoxify AFB1 and AFB2 than *C. citriodora* branch extract (Table 2 & 3). After treatment with *C. citriodora* leaf extract(s), degradation of AFB1 and AFB2 was 99.59% and 96.77% respectively at pH 10 while 95.21% and 92.95% degradation of AFB1 and AFB2 was observed at pH 8. However, *C. citriodora* branch extract showed 83.96% and 69.15% degradation of AFB1 and AFB2 at pH 10. As compared to *C. citriodora* leaf extract(s) at pH 8, degradation of AFB1 and AFB2 by *C. citriodora* branch extract was 78.29% and 66.91% respectively. Distilled water with pH adjusted to 2, 4, 6, 8, and 10 was used as a control. Control data showed that at pH 10, 19.94% of AFB1 and 17.45% of AFB2 was degraded after 72 hr of incubation at 30 °C while 15.88% and 13.05% degradation of AFB1 and AFB2 was observed at pH 8 under same conditions. The percentage of degradation decreases as the pH decreases to neutral or acidic range (Tables 2 & 3). Subsequently, Mendez-Albores *et al*.^21^ also found that aflatoxin florescence, attributed to the coumarin moiety, diminish or even disappear in alkaline treatment. In addition, the similar results were in accordance with the findings of Kannan and Velazhahan^19^ who explored the potential of *Barleria lupulina* leaf extract on detoxification of aflatoxins. Similarly, Hajare *et al*.^20^ found that aflatoxin inactivation using aqueous *Trachyspermum ammi* seeds extract was maximum at pH 10. In the present study, pH 8 was selected for further studies because at this pH *C. citriodora* leaf extracts showed significant (P < 0.05) detoxification of AFB1 and AFB2 and these results were closely comparable with the results obtained at pH 10. Moreover, at high basic pH conditions aflatoxins are known to become unstable and sensitive, therefore to omit this possibility pH 8 was selected which is 100 times less alkaline than pH 10.

### *In Vivo* Detoxification of Aflatoxins in maize samples

*In Vivo* analysis followed a similar trend as that has been recorded in *In Vitro* studies. Data obtained from *In Vivo* studies showed that maximum detoxification of AFB1 and AFB2 in spiked maize samples was carried out by *C. citriodora* leaf extract after 72 hrs of incubation i.e., 91.71% and 88.77% respectively. As compared to *C. citriodora* leaf extract, in *C. citriodora* branch extract 70.26% and 58.84% detoxification of AFB1 and AFB2 was observed in spiked samples (Table 4). Similarly in a study, conducted by Hajare *et al*.^20^, decontamination of spiked corn samples was carried out using ajowan extract. Their findings showed that aflatoxin-contaminated agricultural commodities could be decontaminated using appropriate conditions of temperature and pH along with ajowan inactivation factor.

HPLC chromatograms confirmed that after *C. citriodora* leaf extract treatment, trace amount of aflatoxin was present along with other peaks whose footprints were not found in chromatogram of parent compounds which may be attributed to toxins degradation products (Supplementary Figures 1 & 2).

### Structural characterization of AFB1, AFB2 and their degradation products

To predict molecular formulae as well as elemental composition of degraded products of AFB1 and AFB2 after treatment with *C. citriodora* leaf extract(s), samples were analyzed by mass spectrometer with electrospray ionization (ESI).

Aflatoxin B1 and B2 exhibited good ESI ionization efficiency in the positive ion mode with molecular base ion at m/z 313.17 and m/z 315.17 for protonated adduct [M+ H]^+^ while m/z 335 and m/z 337 for sodium adduct [M+ Na]^+^ respectively. To validate the identity of the parent, these ions were fragmented into daughter ions. Because the sodium adduct did not exhibit specific fragmentation for any compound, the protonated molecule was chosen as the precursor ion for aflatoxins in the product ion scan mode.

### MS/MS analysis of AFB1 and AFB2

**Figure 1:**
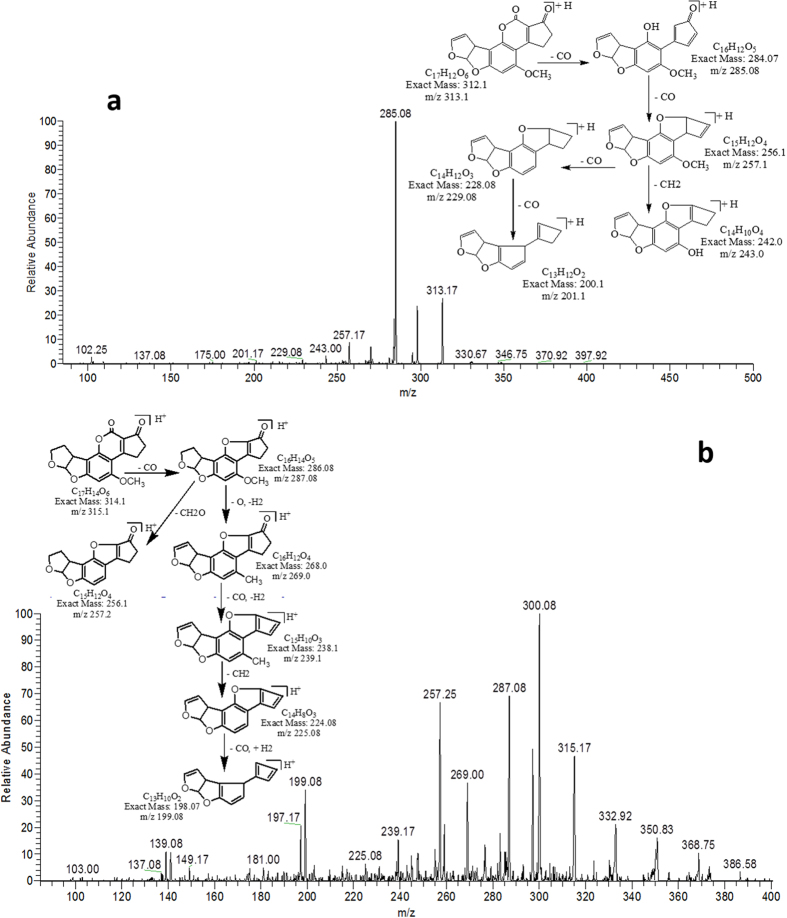
MS/MS Spectra and fragmentation pathway. (**a**) AFB1 and (**b**) AFB2.

MS/MS spectrum of AFB1 showed that continuous loss of carbon monoxide (CO) was the main fragmentation pathway. Methyl and methanol losses occurred on methoxy group located on side chain of benzene. The double bond equivalence (DBE) of AFB1 was 12 (Fig. 1a). However, MS/MS fragmentation pathway of AFB2 revealed that daughter ions were formed by loss of carbon monoxide, oxygen, hydrogen and methyl group (Fig. 1b). The DBE of AFB2 was 11.

The degradation products were identified on the basis of accurate mass measurement of ions and similar fragmentation pathways with AFB1 and AFB2.

**Figure 2:**
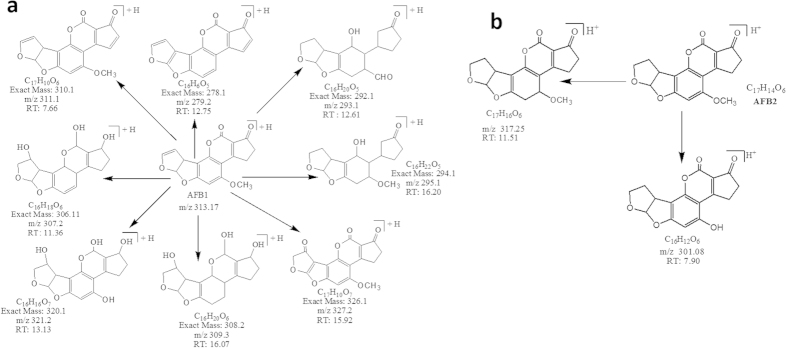
Possible degraded products of (**a**) AFB1 and (**b**) AFB2 after treatment with *C. citriodora* leaf extract(s). Whereas RT indicates the retention time.

Results indicated that after treatment with *C. citriodora* leaf extract(s) ten possible degraded products of AFB1 and AFB2 were formed with structural alteration in parent compound. Structural formulas of possible degraded products of AFB1 and AFB2 are shown in Fig. 2a,b.

### MS/MS analysis for confirmation of degraded products of AFB1

**Figure 3:**
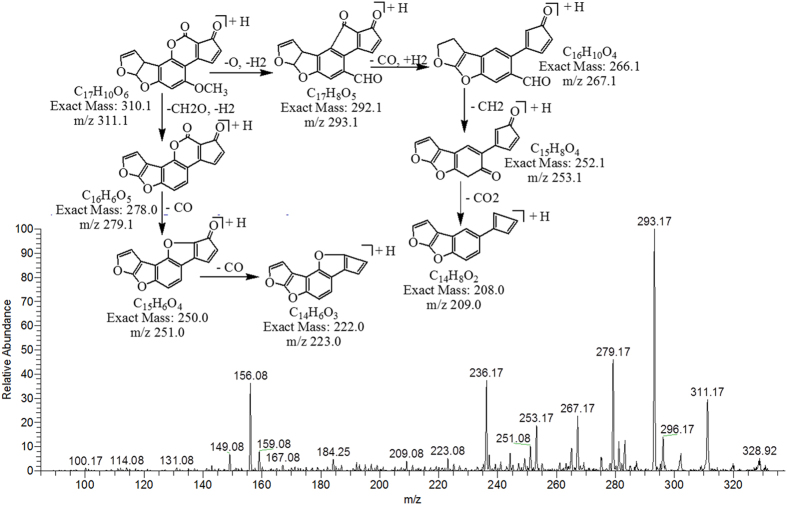
MS/MS Spectra and fragmentation pathway of degraded product with 311.17 m/z.

The degradation product C_17_H_10_O_6_ (with 311.17 m/z) formed by the loss of two hydrogen atoms from AFB1. The DBE of C_17_H_10_O_6_ was 13 which was one more than AFB1. The fragmentation pathway was different from that of AFB1. The precursor ion yielded a series of product ions which are represented by 293.17 [M-H_2_O]^+^, 267.17 [M-CO_2_]^+^, 253.17 [M-C_2_H_2_O_2_]^+^, 279.17 [M-CH_4_O]^+^, 251.08 [M-C_2_H_4_O_2_]^+^, 223.08 [M-C_3_H_4_O_3_]^+^ and 209.08 [M-C_3_H_2_O_4_]^+^ (Fig. 3).

**Figure 4:**
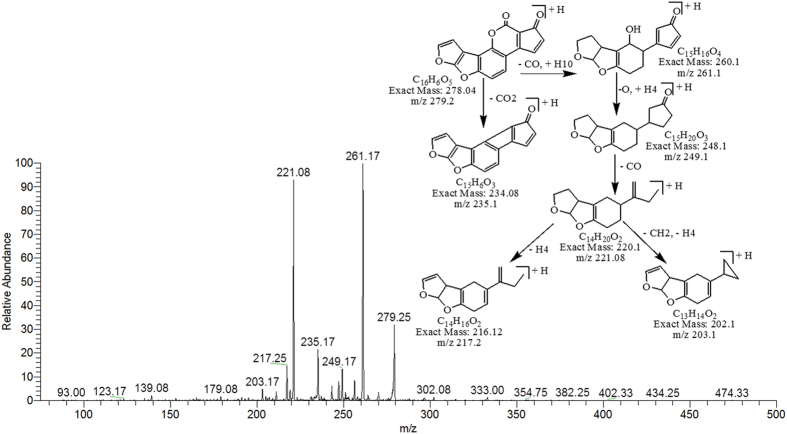
MS/MS Spectra and fragmentation pathway of degraded product with 279.25 m/z.

Degradation product C_16_H_6_O_5_ with ion peak at m/z 279.25 had one less CH_6_O molecule than AFB1 whereas the DBE of C_16_H_6_O_5_ was more than AFB1 i.e., 14. Loss of carbon monoxide (CO), carbon dioxide (CO_2_) and oxygen (O) was the main fragmentation pathway. Removal of double bond occurred due to the addition of hydrogen (Fig. 4).

**Figure 5:**
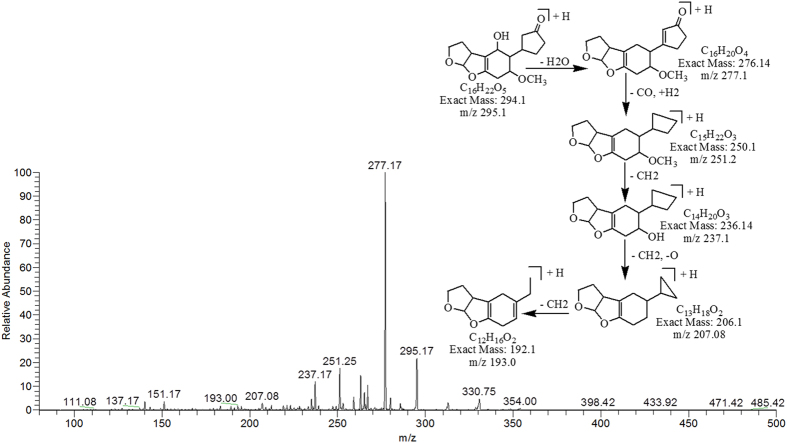
MS/MS Spectra and fragmentation pathway of degraded product with 295.17 m/z.

**Figure 6:**
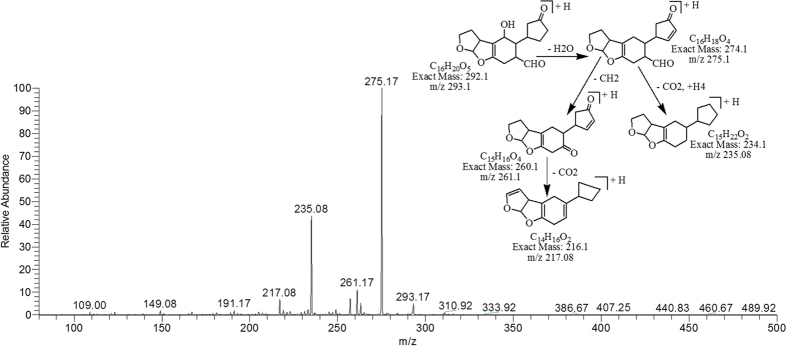
MS/MS Spectra and fragmentation pathway of degraded product with 293.17 m/z.

The degradation products C_16_H_22_O_5_ (with m/z 295.17) and C_16_H_20_O_5_ (with m/z 293.17) were formed due to the loss of carbon monoxide (CO) by the opening of lactone ring and addition of hydrogen atoms to AFB1 molecule. Difference between these two degraded products is only of two hydrogen atoms. The DBE content of both products was same i.e., 6 which was less than AFB1. The fragmentation pathway of both products was different from that of AFB1. The precursor ion C_16_H_22_O_5_ yielded a series of daughter ions i.e., 277.17 [M-H_2_O]^+^, 251.25 [M-CO_2_]^+^, 237.17 [M-C_2_H_2_O_2_]^+^, 207.08 [M-C_3_H_4_O_3_]^+^ and 193.00 [M-C_4_H_6_O_3_]^+^ while product ions formed from parent ion C_16_H_20_O_5_ was represented by 275.17 [M-H_2_O]^+^, 261.17 [M-CH_4_O]^+^, 235.08 [M-CH_2_O_3_]^+^ and 217.08 [M-C_2_H_4_O_3_]^+^ (Figs 5 & 6).

**Figure 7:**
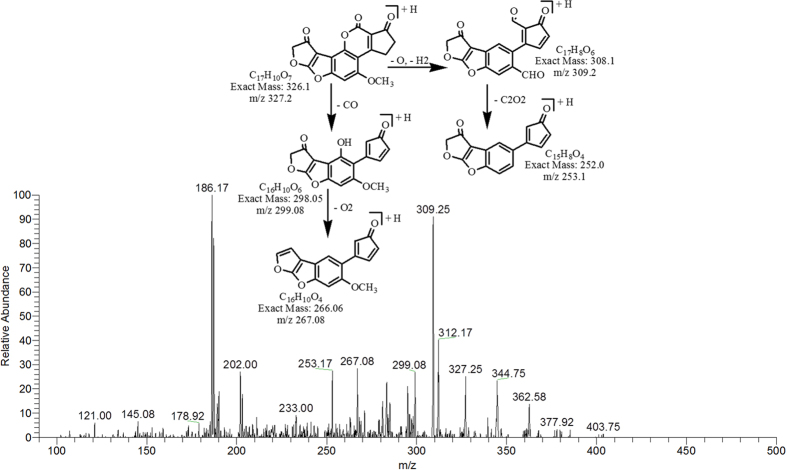
MS/MS Spectra and fragmentation pathway of degraded product with 327.25 m/z.

The degradation product at m/z 327.25 corresponded to molecular formula C_17_H_10_O_7_ was formed by the addition of oxygen atom on the double bond of furan ring on the left side. The DBE of C_17_H_10_O_7_ was one more then AFB1 i.e., 13. Series of products ions formed by the precursor ion represented by 309.25 [M-H_2_O]^+^, 299.08 [M-CO]^+^, 253.17 [M-C_2_H_2_O_3_]^+^ and 267.08 [M-CO_3_]^+^ (Fig. 7).

**Figure 8:**
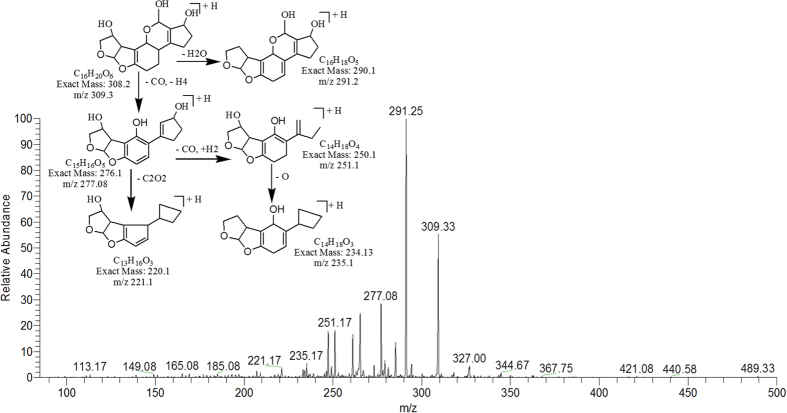
MS/MS Spectra and fragmentation pathway of degraded product with 309.33 m/z.

**Figure 9:**
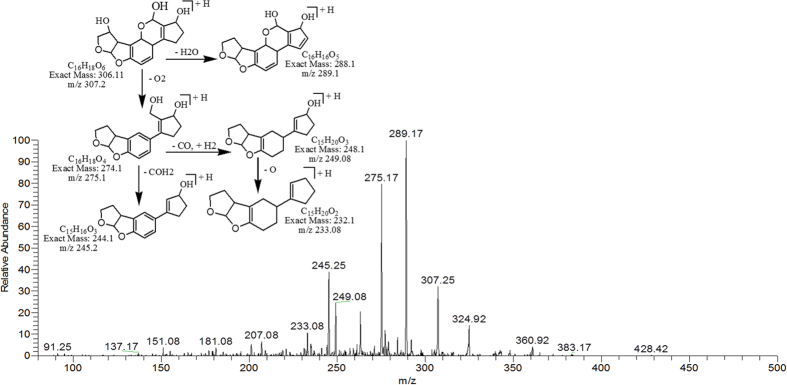
MS/MS Spectra and fragmentation pathway of degraded product with 307.25 m/z.

The degradation products C_16_H_20_O_6_ (with 309.33 m/z) and C_16_H_18_O_6_ (with 307.25) were formed by the loss of carbon monoxide from the side chain of benzene ring and addition of OH group on the double bond of terminal furan ring on left side. While addition of hydrogen atoms occurred on both furan and lactone rings. Difference between them was only of two hydrogen atoms. The DBE of C_16_H_20_O_6_ and C_16_H_18_O_6_ was lower than AFB1 i.e., 7 and 8 respectively. The more detail on fragmentation pathway of these two degraded products are shown in Figs 8 & 9.

**Figure 10:**
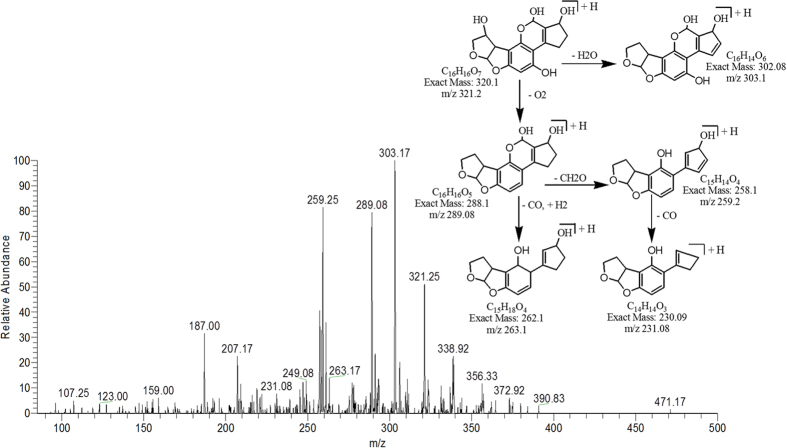
MS/MS Spectra and fragmentation pathway of degraded product with 321.25 m/z.

The degradation product C_16_H_16_O_7_ (with m/z 321.25) had one less carbon atom, as well as four more hydrogen atoms and one more oxygen atom than AFB1. The DBE of C_17_H_20_O_6_ was less than AFB1 i.e., 9. Loss of water, oxygen, and carbon monoxide was the main fragmentation pathway (Fig. 10).

### MS/MS analysis for confirmation of degraded products of AFB2

**Figure 11:**
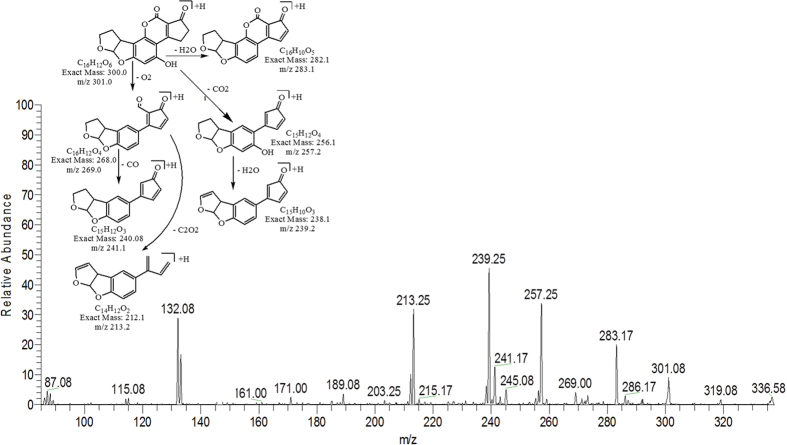
MS/MS spectra and fragmentation pathway of degradation product with 301.08 m/z.

The degradation product C_16_H_12_O_6_ (with m/z 301.08) was formed by the replacement of methoxy group with hydroxyl group on the side chain of benzene ring. The DBE of C_16_H_12_O_6_ was same as that of AFB2. MS/MS spectra showed that the precursor ion yielded product ions at 283.17 [M-H_2_O]^+^, 269.00 [M-O_2_]^+^, 257.25 [M-CO_2_]^+^, 241.17 [M-CO_3_]^+^, 239.25 [M-CH_2_O_3_]^+^ and 213.25 [M-C_2_O_4_]^+^ (Fig. 11).

**Figure 12:**
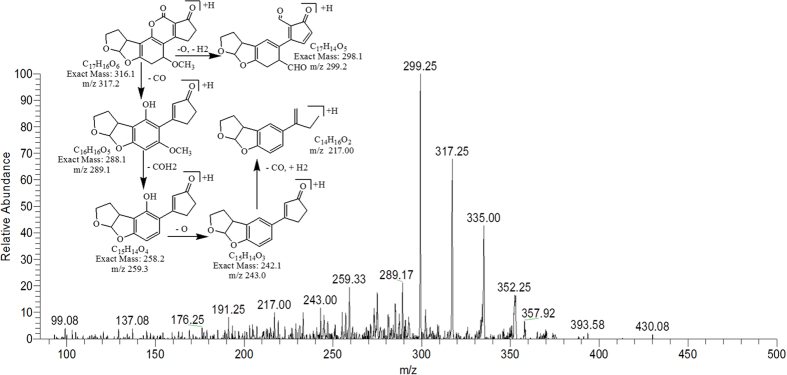
MS/MS spectra and fragmentation pathway of degradation product with 317.25 m/z.

The degradation product C_17_H_16_O_6_ at m/z 317.25 had two more hydrogen atoms and one less DBE than AFB2. Thus, by implication an additional reaction occurred. More detail on fragmentation pathway is shown in Fig. 12.

Several studies have been conducted with the micro-organisms, physical and chemical agents, ultraviolet (UV) rays, Gamma rays and plant products for the aflatoxin detoxification, have shown change of structure of the aflatoxin molecule after detoxification^22^,^23^,^24^,^25^,^26^,^27^,^28^,^29^,^17^,^18^. Aflatoxins have been widely researched for their toxicity by various scientists^1^,^30^,^31^. Their toxicity data showed that aflatoxins have cyclopentene ring and furan moiety in their chemical structure. In AFB1 presence of double bond in the terminal furan ring is key factor for its toxic and carcinogenic activities^29^. In contrast, aflatoxin B2 which have saturated furan ring is hundreds times less carcinogenic^32^. The degraded products of AFB2 may be active but were less potent than that of parent compound. In this present study, data showed that eight possible degradation products of AFB1 were obtained after treatment with *C. citriodora* leaf extract. Among them, 25% products (with m/z 293, 295) were formed after modification of lactone ring and 12% products (with m/z 327) were formed after removal of double bond in terminal furan ring. While in 37% products (with m/z 307, 309, 321) removal of double bond in the terminal furan ring occurred along with modification of lactone ring. Other studies in literature also supported the similar findings. Lee *et al*.^33^ observed that lactone ring plays an important role in fluorescence of aflatoxin molecule. On its cleavage, the molecule becomes non florescent with subsequent significant reduction in toxicity. Velazhahan *et al*.^17^ reported detoxification of aflatoxin G1 by seed extract of Ajowan (*T. ammi*) and suggested the modification of lactone ring structure of AFG1 as mechanism of detoxification. Similar findings were observed by Vijayanandraj *et al*.^18^ after detoxification of aflatoxin B1 by an aqueous extract from leaves of *Adhatoda vasica Nees*. A study conducted by Wang *et al*.^29^ on structure elucidation of radiolytic products of aflatoxin B1 in methanol water solution revealed that in most of radiolytic products addition reaction occurred on the double bond of terminal furan ring, resulting significantly reduced toxicity as compared to that of AFB1. Experiments by Luo *et al*.^34^ showed that aflatoxin B1 can be effectively degraded by using ozone in aqueous system. According to them, due to conjugate addition reaction on the double bond of terminal furan ring for AFB1, the toxicity of degradation products was significantly decreased compared with that of AFB1.

### Assessment of biological toxicity of degraded products

Furthermore, biological toxicity of degraded toxin products were tested by brine shrimps (*Artemia salina*) bioassay. The brine shrimps assay proved to be a convenient system for monitoring biological activity^35^. In this study, the degraded toxin products were incubated with brine shrimp larvae at 26 °C for 24 to 96 hrs. The percentage of mortality was compared with that of control (Table 5). Results indicated that only 12.5–17.5% mortality in brine shrimps larvae was observed after treatment with degraded toxin products. However, mortality level was 82.5–92.5% when larvae were incubated with untreated toxin AFB1 (100 μg L^−1^) and AFB2 (50 μg L^−1^), under same conditions. Percentage of mortality was increased with increase in incubation period. The outcome of study clearly implies that the degraded compounds showed much lower toxicity towards brine shrimps larvae than AFB1 and AFB2 at the tested concentration. Similar findings were also observed by Samuel *et al*.^23^, who worked on detoxification of aflatoxin B1 by *Pseudomonas putida*. He compared the toxicity of treated and untreated AFB1 towards HeLa cells and concluded that degraded products are nontoxic (D1) or much less toxic (D2 and D3) than AFB1 to the cells at the tested concentrations.

## Conclusion

To our knowledge, this is the first report to demonstrate the ability of *Corymbia citriodora* plant extract to detoxify AFB1 and AFB2 in both *In Vitro* and *In Vivo* studies. Maximum detoxification was shown by *C. citriodora* leaf extract(s) at pH 8 and 30 °C temperature after 72 hrs of incubation. Data obtained after structural characterization and biological toxicity assessment of degraded toxin products revealed that their toxicity level is much more less than parent compound. The outcome of study clearly implies the efficient detoxification of aflatoxins by using *C. citriodora* leaf extract(s). These extracts are not only cost effective and environmental safe but also are available to the consumers ubiquitously. The use of these toxin degrading natural products can pave the path for the availability of high quality food products for the end users.

## Methods

### Chemical and reagents

Aflatoxin B1 and B2 purified from *A. flavus* were prepared in laboratory under optimized conditions and compared through HPLC with standard AFB1 and AFB2 purchased from (Sigma-Aldrich, St. Louis, MO, USA). Stock solutions of AFB1 (1000 μg L^−1^) and AFB2 (500 μg L^−1^) were prepared in methanol and stored at 4 °C. The working solutions of AFB1 (100 μg L^−1^) and AFB2 (50 μg L^−1^) were prepared by diluting the stock solution.

### Safety Information

Workers should always wear a face mask, disposable surgical gloves and lab coat when working with the above chemicals and mould cultures.

### Preparation of plant extract

*Corymbia citriodora* leaves and branches were collected from Quaid-e-Azam campus, University of the Punjab, Lahore. Samples were surface-sterilized using 1% sodium hypochlorite for 10 min and washed several times with sterile distilled water. After surface sterilization, aqueous plant extract was prepared by homogenizing 10 g of leaves/branches with 10 mL of sterile distilled water. Homogenate was filtered through muslin cloth and centrifuged at 14,000 rpm for 20 min. Supernatant was sterilized using syringe filter assembly and used for further detoxification studies.

### *In Vitro* Toxin inactivation assay

For detoxification studies, 50 μL of working solution containing (100 μg L^−1^) AFB1 and (50 μg L^−1^) AFB2 was mixed with 250 μL of *C. citriodora* plant extracts and incubated for various intervals of time. After incubation period, reaction was terminated by addition of equal volume of chloroform. The residual toxin was extracted by vortexing the mixture thoroughly. Chloroform fraction was separated by low speed centrifugation. After centrifugation, organic phase was withdrawn, evaporated to dryness under gentle stream of nitrogen and re-dissolved in methanol. Control consisted of 50 μL of toxin in 250 μL of water and was incubated under same conditions.

### Determination of optimal pH for toxin detoxification by plants extract (*In Vitro*)

The optimal pH was determined by modifying the original pH of the *C. citriodora* aqueous extracts in the range of 2.0 to 10.0 (adjusted using either 1 N HCl or 1 N NaOH) and then assayed for toxin detoxification activity. Distilled water with same pH range as well as untreated extract was used as control.

### Determination of optimal temperature and incubation time period for toxin detoxification by plants extract (*In Vitro*)

For assessing optimum temperature and incubation period, *C. citriodora* extracts were incubated with toxins at 25 °C, 30 °C, 35 °C, 40 °C, 45 °C, 50 °C, 55 °C and 60 °C for 3, 6, 12, 24, 48 and 72 hr respectively. After incubation, the toxin content in the reaction mixture was determined as described above.

### Detoxification of maize samples using plant extracts (*In Vivo* studies)

For this purpose, maize samples were spiked with aflatoxins (B1 100 μg L^−1^ & B2 50 μg L^−1^) and incubated with *C. citriodora* aqueous extract under optimized conditions of pH, temperature and time intervals. At the end of the treatment, treated toxins extraction was performed according to the following method. Maize samples were extracted with water–methanol (15:85 v/v) and incubated on shaking water bath for two hours. After incubation, the extracts were filtered through filter paper (Whatman, Inc., Clifton, NJ, USA). Immunoaffinity columns were conditioned with double distilled water. Then, the filtrate was passed through the column in a solid phase extraction assembly. Toxins were slowly eluted from the column with 1 mL methanol in a glass vial. The residual toxin was qualitatively and quantitatively analyzed by TLC and HPLC respectively. Controls consist of untreated maize sample, sample with toxin without *C. citriodora* extract, sample with *C. citriodora* extract without toxin while replicates are biological.

### Detection and Quantification of treated toxin

The detection and qualification of residual toxin was determined by thin layer chromatography (TLC). Twenty microliters of chloroform methanol fraction of treated and control samples were spotted on 0.25 mm silica gel 60F_254_ (20 × 20 cm, Merck) TLC plate and developed in chloroform: acetone (92:8 v/v). The developed plates were viewed under UV light at 365nm.

Quantitative analysis of treated and untreated toxin was done by using High Performance Liquid Chromatography (HPLC) after derivatization. A HPLC system (Agilent 1100 series, Agilent Technologies, Santa Clara, CA, USA) with a reversed- phase C18 column (Merck, Darmstadt, Germany) and a fluorescence detector was used for quantification. Mobile phase consisting of water: methanol: acetonitrile in the volume ratio 60:20:20 at a flow rate of 1 mL/min was applied and aflatoxin was detected at excitation and emission wavelengths of 360 nm and 440 nm respectively. For HPLC method validation, calibration curves were drawn using a series of calibration solutions in methanol. Each standard solution was chromatographed in duplicate. Further, identification of degraded toxin metabolites was carried out by mass spectral studies.

### LCMS analysis of degraded toxin

Degraded toxin products were analyzed by using surveyor LC system equipped with mass spectrophotometer and PDA plus detectors (Thermo Fisher Scientific). System was validated with known standards individually and in mixture form. All analysis were performed in triplicate using luna phenomenex C_18_ column (150 × 4.6 mm, 3 μm), in isocratic mode. Following are the LC-MS conditions for Aflatoxins. Injection volume was 10 μL. Mobile phase consisted of Methanol: Acetonitrile: Water (22.5: 22.5: 55.0 v/v). Column temperature was maintained at 30 °C. The total operation time was 25 min with the flow rate of 0.5 mL min^−1^. MS conditions were as follows: capillary temperature was 335 °C, sheath gas flow and Auxillary gas flow was 20 L min^−1^ and 4 L min^−1^ respectively. Source voltage, capillary voltage and tube lens voltage was 5 KV, 49 V and 120 V respectively.

### ESI–MS/MS Conditions for Aflatoxins through direct insertion pump

Mass spectrometery/ Mass spectrometery was performed on a Thermo Scientific LTQ XL System fitted with electrospray ionization (ESI) source operating in positive ionization mode with optimum conditions set as follows: capillary voltage to 49.0 V, source voltage to 5.0 KV, Tube lens voltage to 110 V, and capillary temperature to 275 °C. Sheath and auxillary gas flow were adjusted to get stable spray i.e., 3 L min^−1^ and 0.4 L min^−1^ respectively. Data was collected in positive mode within the range of 100 m/z to 500 m/z. The final identification of an unknown compound was then performed based on the accurate measurement of mass of parent ions and fragments, as well as other useful MS/MS spectrum information.

### Testing biological toxicity of degraded products

The biological toxicity of degraded toxin products was tested by brine shrimps (*Artemia salina*) bioassay. The procedure for bioassay generally followed the method developed by Solis^36^, with some modifications. Brine shrimps dry eggs were procured from local market. 100–200 mg of shrimps eggs were hatched in artificial sea water (34 g sea salt/ L of deionized water) by incubation under 60 W lamp, providing direct light and warmth (26 °C). Throughout hatching period, the same conditions of light sensitivity and temperature were maintained. After an incubation period, the hatched nauplii were separated from shells and transferred to fresh sea water.

The volume of 300 μl of treated and untreated AFB1 (100 μg L^−1^) & AFB2 (50 μg L^−1^) solution was added to 96 well plate separately and dried overnight. After complete evaporation of solvent, toxins were redissolved in 200 μL of sea water. After that, 200 μL of sea water containing 40–45 organisms were pipetted into each well, resulting in a final volume of 400 μL and incubated for 24–96 hrs at 26 °C. Mortality was determined by counting the immobile (dead) larvae under stereoscope microscope. Toxicity of each solution was evaluated in triplicate.

### Statistical analysis

Results obtained in various experiments were subjected to statistical analysis by using DSSTAT software. Data were analyzed by analysis of Variance (ANOVA) and differences among the means were determined for significance at P ≤ 0.05 using Tukey’s multiple range test.

## Additional Information

**How to cite this article**: Iram, W. *et al*. Mass spectrometric identification and toxicity assessment of degraded products of aflatoxin B1 and B2 by *Corymbia citriodora* aqueous extracts. *Sci. Rep*. **5**, 14672; doi: 10.1038/srep14672 (2015).

## Supplementary Material

Supplementary Information

## Figures and Tables

**Table 1:** Effect of Temperature on Toxin Detoxification by *C. cirtidora* plant extracts.

CONTROL	Temp (^O^C)	DETOXIFICATION (%) AFB1						DETOXIFICATION (%) AFB2					
		3 hr	6 hr	12 hr	24 hr	48 hr	72 hr	3 hr	6 hr	12 hr	24 hr	48 hr	72 hr
TOXIN	25	0.25^d^	0.71^cd^	1.45^bc^	1.71^b^	1.94^b^	2.86^a^	0.12^c^	0.53^bc^	0.72^abc^	0.84^abc^	1.02^ab^	1.44^a^
	30	0.80^d^	0.80^d^	1.88^cd^	2.30^bc^	3.07^ab^	3.81^a^	0.17^c^	0.68^bc^	0.79^bc^	0.99^ab^	1.16^ab^	1.66^a^
	35	1.21^c^	2.54^bc^	2.54^bc^	3.01^abc^	3.80^ab^	4.49^a^	0.23^c^	0.82^bc^	0.87^bc^	1.14^b^	1.31^ab^	1.88^a^
	40	2.21^b^	3.53^ab^	3.87^ab^	4.33^a^	4.46^a^	5.15^a^	0.28^c^	0.94^bc^	0.97^bc^	1.29^b^	1.46^ab^	2.11^a^
	45	3.20^b^	4.53^ab^	5.13^a^	5.19^a^	5.66^a^	5.82^a^	0.33^c^	1.02^bc^	1.12^b^	1.44^b^	1.61^b^	2.33^a^
	50	4.19^b^	5.52^ab^	5.79^ab^	6.48^a^	6.51^a^	6.98^a^	0.38^c^	1.09^bc^	1.27^b^	1.59^b^	1.76^b^	2.55^a^
	55	5.18^c^	6.45^bc^	6.51^bc^	7.14^ab^	7.84^ab^	8.30^a^	0.43^d^	1.16^cd^	1.42^bc^	1.73^bc^	1.91^b^	2.78^a^
	60	6.18^c^	7.11^c^	7.50^c^	7.80^bc^	9.16^ab^	9.63^a^	0.49^d^	1.24^cd^	1.57^bc^	1.88^bc^	2.06^b^	3.00^a^
TOXIN + WATER	25	0.28^b^	1.48^b^	2.72^ab^	3.38^a^	3.35^a^	3.36^a^	0.29^c^	0.47^c^	1.32^bc^	1.47^abc^	2.25^ab^	2.46^a^
	30	1.10^c^	2.64^bc^	3.23^ab^	3.56^ab^	3.97^ab^	4.19^a^	0.36^c^	1.14^bc^	1.56^bc^	2.04^ab^	2.41^ab^	3.41^a^
	35	2.44^c^	3.44^bc^	4.80^ab^	5.09^a^	5.49^a^	5.85^a^	1.20^c^	1.23^c^	2.26^b^	2.73^ab^	2.87^ab^	3.34^a^
	40	3.76^c^	4.76^bc^	6.13^ab^	6.48^a^	6.74^a^	6.84^a^	1.98^c^	2.69^bc^	3.37^ab^	3.40^ab^	3.71^a^	3.84^a^
	45	5.08^c^	6.08^bc^	7.45^ab^	7.47^ab^	7.83^a^	8.40^a^	2.72^c^	3.92^b^	4.09^ab^	4.18^ab^	4.49^ab^	4.96^a^
	50	6.41^c^	7.41^bc^	8.46^ab^	8.77^ab^	8.82^ab^	10.05^a^	3.46^b^	4.44^b^	4.46^b^	5.60^a^	5.67^a^	6.07^a^
	55	7.73^c^	8.73^bc^	9.46^bc^	9.82^b^	10.10^b^	11.71^a^	4.21^b^	4.83^b^	4.96^b^	6.72^a^	7.15^a^	7.19^a^
	60	9.05^c^	10.05^bc^	10.45^bc^	10.81^bc^	11.42^b^	13.36^a^	4.95^b^	5.20^b^	5.48^b^	7.84^a^	8.31^a^	8.64^a^
**TREATMENTS**													
TOXIN + CORYMBIA LEAF	25	54.51^d^	57.22^d^	63.80^cd^	70.38^bc^	76.95^ab^	81.68^a^	71.37^c^	74.35^bc^	77.32^abc^	78.81^ab^	80.30^ab^	81.04^a^
	30	60.41^d^	64.84^d^	69.26^cd^	73.69^bc^	78.12^ab^	82.55^a^	72.86^c^	76.08^bc^	79.55^abc^	81.04^ab^	82.03^ab^	83.27^a^
	35	63.95^e^	68.49^de^	73.04^cd^	77.58^bc^	82.13^ab^	86.67^a^	74.35^b^	78.31^ab^	81.79^a^	82.53^a^	83.52^a^	84.76^a^
	40	66.38^e^	70.00^de^	74.97^cd^	79.94^bc^	84.91^ab^	89.88^a^	75.83^b^	80.55^ab^	84.02^a^	84.02^a^	85.01^a^	86.25^a^
	45	74.63^d^	78.83^d^	83.03^c^	87.22^b^	91.42^a^	93.72^a^	77.32^b^	82.78^ab^	86.25^a^	85.51^a^	86.50^a^	87.74^a^
	50	80.62^e^	84.88^d^	89.14^c^	93.40^b^	94.68^ab^	96.82^a^	78.81^b^	85.01^ab^	88.48^a^	86.99^a^	87.99^a^	89.23^a^
	55	87.17^e^	91.91^d^	93.79^c^	94.94^bc^	96.49^ab^	97.94^a^	80.30^b^	87.24^ab^	90.72^a^	88.48^a^	89.48^a^	90.72^a^
	60	94.11^d^	96.16^c^	97.89^b^	98.58^b^	99.38^a^	99.56^a^	81.79^b^	89.48^a^	89.97^a^	90.96^a^	91.20^a^	92.20^a^
TOXIN + CORYMBIA BRANCH	25	33.57^d^	38.02^cd^	42.93^cd^	51.45^bc^	60.68^ab^	67.94^a^	37.90^e^	42.36^de^	47.57^cd^	51.29^bc^	55.01^ab^	59.10^a^
	30	38.93^c^	41.89^c^	45.01^c^	52.19^bc^	63.06^ab^	69.13^a^	39.38^e^	44.10^de^	49.80^cd^	53.52^bc^	56.75^ab^	60.96^a^
	35	43.10^c^	46.35^c^	49.18^c^	55.76^bc^	66.33^ab^	71.81^a^	40.87^d^	46.33^cd^	52.03^bc^	55.01^b^	58.23^ab^	62.45^a^
	40	50.30^c^	53.80^bc^	56.03^bc^	62.91^b^	73.78^a^	78.96^a^	42.36^d^	48.56^cd^	54.27^bc^	56.50^b^	59.72^ab^	63.94^a^
	45	51.78^c^	57.37^bc^	59.30^bc^	64.10^b^	74.37^a^	82.53^a^	43.85^d^	50.79^cd^	56.50^bc^	57.99^b^	61.21^ab^	65.43^a^
	50	52.16^d^	55.95^d^	59.90^cd^	64.40^c^	74.67^b^	83.72^a^	45.34^d^	53.03^c^	58.73^bc^	59.47^b^	62.70^ab^	66.91^a^
	55	54.46^d^	58.71^cd^	62.88^cd^	67.67^bc^	75.59^b^	84.02^a^	46.82^d^	55.26^c^	60.96^bc^	60.96^bc^	64.19^ab^	68.40^a^
	60	56.55^d^	60.20^cd^	63.18^cd^	68.27^bc^	76.56^b^	85.21^a^	48.31^d^	57.49^c^	62.45^bc^	63.19^bc^	65.67^ab^	69.89^a^

Data were analyzed by analysis of Variance (ANOVA). Values with different alphabetic letters indicate significant differences (P < 0.05) among tested plant extracts as determined by Tukey’s Multiple Range test.

**Table 2:** Effect of pH on Aflatoxin B1 Detoxification by *C. citriodora* leaf and branch extracts.

CONTROL	PH	3 hr		6 hr		12 hr		24 hr		48 hr		72 hr	
		Toxin recovery(μg L^−1^)	D %	Toxin recover(μg L^−1^)	D %	Toxin recover(μg L^−1^)	D %	Toxin recover(μg L^−1^)	D %	Toxin recover(μg L^−1^)	D %	Toxin recovery(μg L^−1^)	D %
Toxin AFB1		99.19^r^	0.81	99.12^r^	0.88	98.20^r^	1.80	97.70^r^	2.30	96.93^qr^	3.07	96.19^o–r^	3.81
Toxin + H_2_O	PH 2	96.82^pqr^	3.18	95.38^n–r^	4.62	92.19^k–p^	7.81	90.42^g–l^	9.58	89.76^e–l^	10.24	87.37^d–j^	12.63
Toxin + H_2_O	PH 4	96.16^o–r^	3.84	95.27^n–r^	4.73	90.75^h–m^	9.25	88.44^d–l^	11.56	87.00^d–i^	13.00	86.12^c–h^	13.88
Toxin + H_2_O	PH 6	94.94^m–r^	5.06	92.52^l–q^	7.48	90.20^f–l^	9.80	88.00^d–l^	12.00	86.01^c–g^	13.99	85.57^b–f^	14.43
Toxin + H_2_O	PH 8	91.85^j–o^	8.15	89.87^e–l^	10.13	87.66^d–k^	12.34	85.31^b–e^	14.69	84.12^a–d^	15.90	84.10^a–d^	15.88
Toxin + H_2_O	PH 10	91.44^i–n^	8.56	87.78^d–k^	12.22	85.26^b–e^	14.74	82.34^abc^	17.66	81.19^ab^	18.81	80.06^a^	19.94
Toxin + H_2_O	WpH	98.90^r^	1.10	97.36^r^	2.64	96.77^pqr^	3.23	96.44^o–r^	3.56	96.03^o–r^	3.97	95.81^n-r^	4.19
TREATMENT													
Corymbia Leaf + AFB1 Corymbia	PH 2	38.92^h–u^	61.08	34.16^c–u^	65.84	29.40^a–t^	70.60	24.63^a–o^	75.37	19.87^a–l^	80.13	15.10^a–j^	84.90
	PH 4	31.67^b–u^	68.33	27.45^a–r^	72.55	23.24^a–n^	76.76	19.02^a–l^	80.98	14.81^a–j^	85.19	10.59^a–i^	89.41
	PH 6	26.53^a–q^	73.47	22.66^a–n^	77.34	18.79^a–l^	81.21	14.92^a–j^	85.08	11.05^a–i^	88.95	7.17^a–e^	92.83
	PH 8	22.95^a–n^	77.05	18.36^a–l^	81.64	13.77^a–j^	86.23	9.17^a–g^	90.83	7.70^a–f^	92.30	4.79^abc^	95.21
	PH 10	15.49^a–j^	84.51	12.47^a–j^	87.53	9.46^a–h^	90.54	6.44^a–d^	93.56	3.43^ab^	96.57	0.41^a^	99.59
	WpH	39.59^i–u^	60.41	35.16^d–u^	64.84	30.74^b-t^	69.26	26.31^a–q^	73.69	21.88^a–m^	78.12	17.45^a–k^	82.55
Branch + AFB1	PH 2	57.42^stu^	42.58	56.26^r–u^	43.74	53.97^o–u^	46.03	50.27^m–u^	49.73	41.93^j–u^	58.07	39.94^i–u^	60.06
	PH 4	54.79^p–u^	45.21	53.32^o–u^	46.68	51.50^n–u^	48.50	49.75^m–u^	50.25	46.18^k–u^	53.82	38.14^g–u^	61.86
	PH 6	41.68^j–u^	58.32	37.54^g–u^	62.46	34.21^c–u^	65.79	31.50^b–u^	68.50	25.54^a–q^	74.46	22.92^a–n^	77.08
	PH 8	36.65^e–u^	63.35	35.46^d–u^	64.54	32.00^b–u^	68.00	29.91^a–t^	70.09	25.31^a–p^	74.69	21.71^a–m^	78.29
	PH 10	34.74^d–u^	65.26	31.46^b–u^	68.54	28.60^a–t^	71.40	28.01^a–s^	71.99	21.75^a–m^	78.25	16.04^a–j^	83.96
	WpH	61.07^u^	38.93	58.11^tu^	41.89	47.81^q–u^	45.01	47.81^l–u^	52.19	36.94^f–u^	63.06	30.87^b–t^	69.13

Data were analyzed by analysis of Variance (ANOVA). Values with different alphabetic letters indicate significant differences (P < 0.05) as determined by Tukey’s Multiple Range test. D%: Detoxification %age. WpH: without pH.

**Table 3:** Effect of pH on Aflatoxin B2 Detoxification by *C. citriodora* leaf and branch extracts.

CONTROL	PH	3 hr		6 hr		12 hr		24 hr		48 hr		72 hr	
		Toxin recovery(μg L^−1^)	D %	Toxin recovery(μg L^−1^)	D %	Toxin recovery(μg L^−1^)	D %	Toxin recovery(μg L^−1^)	D %	Toxin recovery(μg L^−1^)	D %	Toxin recovery(μg L^−1^)	D %
Toxin AFB2		49.91^n^	0.17	49.66^lmn^	0.68	49.60^k–n^	0.79	49.50^h–n^	0.99	49.42^g–n^	1.16	49.17^d–l^	1.66
Toxin + H_2_O	PH 2	49.87^n^	1.26	49.83^mn^	1.74	49.60^k–n^	4.03	49.58^j–n^	4.15	49.57^i–n^	4.22	49.42^g–n^	5.84
Toxin + H_2_O	PH 4	49.72^lmn^	2.78	49.67^lmn^	3.34	49.32^e–n^	6.83	49.29^d–n^	7.15	49.24^d–m^	7.57	49.11^d–l^	8.92
Toxin + H_2_O	PH 6	49.36^f–n^	6.40	49.35^f–n^	6.51	49.02^c–k^	9.85	48.97^c–i^	10.34	48.91^c–h^	10.87	48.91^c–h^	10.89
Toxin + H_2_O	PH 8	49.22^d–m^	7.83	48.86^b–g^	11.36	48.83^a–g^	11.71	48.72^a–e^	12.76	48.70^a–e^	12.97	48.69^a–d^	13.05
Toxin + H_2_O	PH 10	48.77^a–f^	12.35	48.46^abc^	15.38	48.45^abc^	15.46	48.44^abc^	15.56	48.28^ab^	17.23	48.25^a^	17.54
Toxin + H_2_O	WpH	49.82^mn^	0.36	49.43^g–n^	1.14	49.22^d–m^	1.56	48.98^c–j^	2.04	48.80^a–f^	2.41	48.29^ab^	3.41
TREATMENT													
Corymbia Leaf + AFB2	PH 2	16.50^e–s^	67.00	15.01^d–q^	69.98	13.52^c–p^	72.96	12.03^a–n^	75.93	10.55^a–k^	78.91	9.99^a–j^	80.02
	PH 4	15.06^d–q^	69.88	13.73^c–p^	72.55	12.65^b–o^	74.71	12.01^a–n^	75.99	11.08^a–l^	77.83	9.96^a–j^	80.07
	PH 6	14.69^d–p^	70.6	13.57^c–p^	72.86	11.52^a–m^	76.95	11.15^a–l^	77.69	10.04^a–j^	79.93	9.29^a–h^	81.41
	PH 8	12.83^b–o^	3	11.71^a–n^	76.58	9.11^a–h^	81.79	7.99^a–f^	84.02	7.62^a–e^	84.76	3.53^abc^	92.95
	PH 10	9.48^a–i^	81.04	7.99^a–f^	84.02	4.64^a–d^	90.72	3.28^abc^	93.44	2.41^ab^	95.18	1.62^a^	96.77
	WpH	13.57^c–p^	72.86	11.96^a–n^	76.08	10.22^a–k^	79.55	9.48^a–i^	81.04	8.98^a–h^	82.03	8.36^a–g^	83.27
Corymbia Branch + AFB2	PH 2	33.28^y^	33.43	31.67^xy^	36.66	29.94^v–y^	40.13	29.19^u–y^	41.62	28.70^u–y^	42.61	26.59^s–y^	46.82
	PH 4	31.80^xy^	36.41	29.44^u–y^	41.12	26.59^s–y^	46.82	24.73^q–y^	50.54	23.12^o–y^	53.77	21.01^k–x^	57.99
	PH 6	29.56^u–y^	40.87	27.21^s–y^	45.58	24.36^p–y^	51.29	22.50^n–y^	55.01	20.88^j–x^	58.23	18.78^f–u^	62.45
	PH 8	27.33^s–y^	45.34	24.98^q–y^	50.05	22.12^m–x^	55.75	20.26^i–w^	59.47	18.65^f–u^	62.70	16.54^e–s^	66.91
	PH 10	26.22^r–y^	47.57	23.86^p–y^	52.28	21.01^k–x^	57.99	19.15^g–v^	61.71	17.54^e–t^	64.93	15.43^d–r^	69.15
	WpH	30.31^wxy^	39.38	27.95^t–y^	44.10	25.10^q–y^	49.80	23.24^o–y^	53.52	21.63^l–x^	56.75	19.52^h–w^	60.96

Data were analyzed by analysis of Variance (ANOVA). Values with different alphabetic letters indicate significant differences (P < 0.05) as determined by Tukey’s Multiple Range test. D%: Detoxification %age. WpH: without pH change.

**Table 4:** *In Vivo* detoxification of AFB1 and AFB2 at pH 8 and 30 °C after 72 hrs of incubation.

CONTROL	Toxin recovery(μg L^−1^)	
	AFB1	AFB2
Unspiked maize	0.49	0.33
Unspiked maize + *C. citriodora* leaf extract(s)	0.00	0.00
Unspiked maize + *C. citriodora* branch extract	0.00	0.00
Spiked maize with AFB1(100 ng/ml) and AFB2 (50 ng/ml)	97.30	47.65
**TREATMENTS**		
Spiked maize with toxin + *C. citriodora* leaf extract (s)	8.29	5.62
Detoxification (%)	91.71	88.77
Spiked maize with toxin + *C. citriodora* branch extract	29.74	20.58
Detoxification (%)	70.26	58.84

Values are mean of three replicates. Data were analyzed by analysis of Variance (ANOVA.

**Table 5:** Percent mortality of brine shrimps (*Artemia salina*) larvae at 26 °C after treatment with toxin (AFB1 & AFB2) detoxified with *E. citriodora* leaves extract at various incubation periods.

TREATMENTS	Incubation period (hrs.)	No. of living shrimps	No. of dead shrimps	% Mortality
**CONTROL**				
Sea water + shrimps	24	40	0	0
	48	40	0	0
	72	40	0	0
	96	39	1	2.5
Methanol + shrimps	24	38	1	2.5
	48	38	2	5
	72	37	3	7.5
	96	36	3	7.5
Untreated toxins + shrimps	24	7	33	82.5
	48	5	35	87.5
	72	4	36	90
	96	3	37	92.5
**TREATMENT**				
Treated toxin with *E. citriodora* leaves extract + shrimps	24	35	5	12.5
	48	34	6	15
	72	34	6	15
	96	33	7	17.5

Sea water was taken as a control. Other Controls consist of Methanol and untreated toxin AFB1 (100 μg L^−1^) and AFB2 (50 μg L^−1^) dried and redissolved in sea water. Values are mean of three replicates. Data were analyzed by analysis of Variance (ANOVA).
