# Supplementary material for: Mass spectrometric identification and toxicity assessment of degraded products of aflatoxin B1 and B2 by Corymbia citriodora aqueous extracts
Source: Sci Rep. 2015 Oct 1;5:14672. doi: 10.1038/srep14672 (PMC4589780; doi:10.1038/srep14672)
Supplement: Supplementary Information [file srep14672-s1.pdf]

Mass spectrometric identification and toxicity assessment of degraded products of aflatoxin B1 and B2 by *Corymbia citriodora* aqueous extracts

Wajiha Iram [a], Tehmina Anjum [a *], Mazhar Iqbal [b], Abdul Ghaffar [c], Mateen Abbas[d]

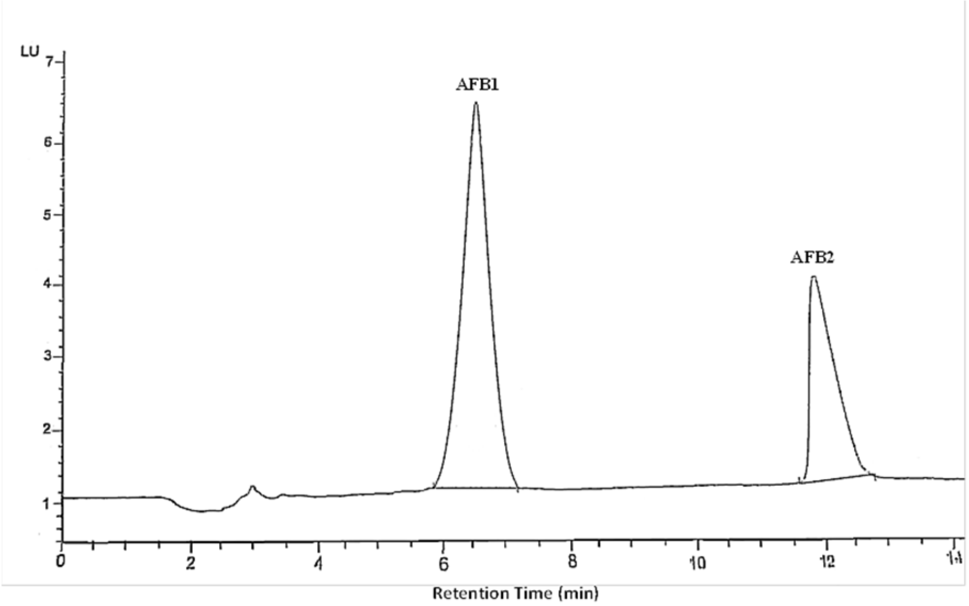

Supplementary Figure 1 HPLC chromatogram of untreated AFB1 and AFB2 with retention time of 6.46 and 12.63 min respectively.

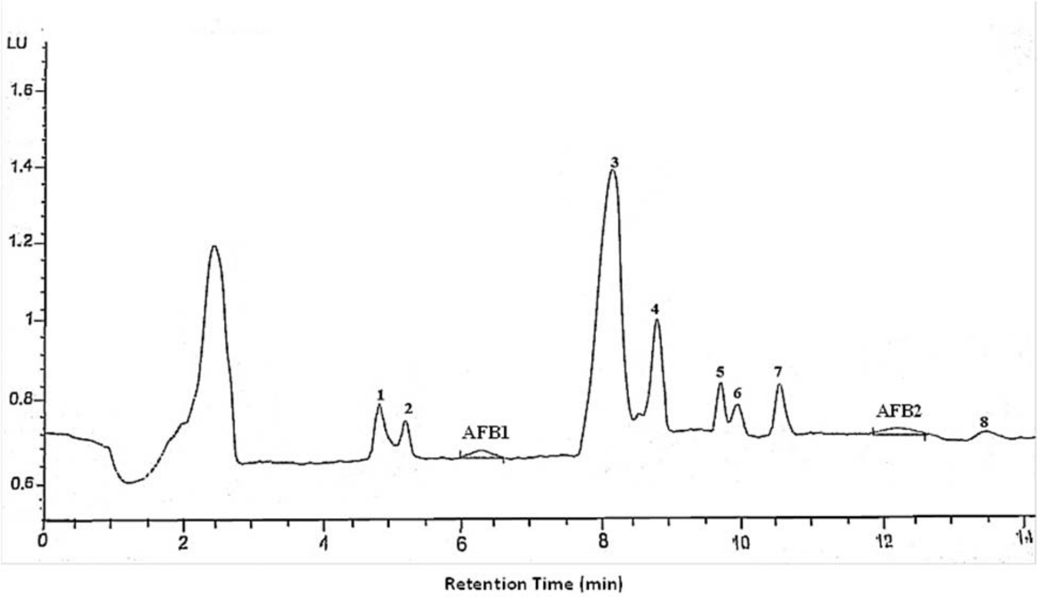

Supplementary Figure 2 HPLC chromatogram of AFB1 and AFB2 after treatment with aqueous extract of *Corymbia citriodora* leaves under optimized conditions (*In Vivo*). Peaks denoted by numbers appeared after treatment whose footprint was not found in untreated AFB1 and AFB2 chromatogram.
